# Neuroprotective Potential of *Tetraselmis chuii* Compounds: Insights into Blood–Brain Barrier Permeability and Intestinal Transport

**DOI:** 10.3390/ph18050629

**Published:** 2025-04-26

**Authors:** Melis Cokdinleyen, Alberto Valdés, Huseyin Kara, Elena Ibáñez, Alejandro Cifuentes

**Affiliations:** 1 Laboratory of Foodomics, Institute of Food Science Research, CIAL, CSIC, Nicolás Cabrera 9, 28049 Madrid, Spain; melis.cokdinleyen@cial.uam-csic.es (M.C.); a.cifuentes@csic.es (A.C.); 2 Department of Chemistry, Faculty of Sciences, Selçuk University, Ardicli, Ismetpasa Cad, Selçuklu, 42250 Konya, Turkey; hkara@selcuk.edu.tr

**Keywords:** Alzheimer’s disease, blood–brain barrier, intestinal permeability, neuroprotection, pressurized liquids extraction

## Abstract

**Background/Objectives:** Alzheimer’s disease (AD) is the most common type of dementia, characterized by complex processes such as neuro-inflammation, oxidative damage, synaptic loss, and neuronal death. Carotenoids are among the potential therapeutic molecules that have attracted attention due to their neuroprotective properties, but their efficacy is limited mainly by their capacity to cross the blood–brain barrier (BBB). **Results:** The results showed that *T. chuii* extracts could protect neuronal cells from neurotoxic damage, especially against L-glutamate and H_2_O_2_. Moreover, the BBB permeability and the intestinal transport analyses revealed that fucoxanthinol, crocoxanthin, diatoxanthin, neoxanthin, violaxanthin, and prasinoxanthin have diverse permeabilities depending on the incubation time and the cell model used. Fucoxanthinol was the carotenoid with the highest and similar permeability in HBMEC cells (4.41%, 5.13%, and 18.94% at 2, 4, and 24 h, respectively) and Caco-2 cells (7.01%, 8.63%, and 18.36% at the same times), while crocoxanthin, diatoxanthin, and neoxanthin showed different kinetics. **Methods:** The neuroprotective potential of two extracts obtained from *Tetraselmis chuii* microalga were evaluated against A*β*1-42-, L-glutamate-, and H_2_O_2_-induced toxicities in SH-SY5Y cells. In addition, the BBB permeability and the intestinal transepithelial transport of the main carotenoids present in the extracts were evaluated and compared using two cell culture models, HBMEC and Caco-2 cells. For that aim, the transport of the bioactive molecules across the barriers was evaluated using UHPLC-q-TOF-MS after 2, 4, and 24 h of incubation. **Conclusions:** These findings indicate that *T. chuii* is a promising natural source of bioactive compounds to develop functional foods against neurodegenerative diseases.

## 1. Introduction

Demographic change caused by the aging population plays a fundamental role in the increase of neurodegenerative disorders, especially Alzheimer’s disease (AD). Currently, approximately 35 million people worldwide are affected by AD, and this number is expected to reach 65 million by 2030 [1]. AD is a multifactorial neurodegenerative disorder, including amyloid-beta (A*β*) accumulation, tau protein hyperphosphorylation, neuro-inflammation, and oxidative stress [2]. However, current treatments are aimed at relieving symptoms rather than correcting the underlying pathological mechanisms [3]. Additionally, the blood–brain barrier (BBB), a dynamic barrier between the central nervous system (CNS) and the systemic circulation, makes it difficult for bioactive substances to pass into the brain, thus preventing 98% of neurotherapeutic agents from reaching their target [4,5].

Nowadays, natural products are attracting attention as potential treatment options, given the limited efficacy and side effects of the actual treatments [6]. In this regard, microalgae are known to contain a variety of beneficial metabolites, such as pigments (carotenoids), polyunsaturated fatty acids, phenolic compounds, alkaloids, and polysaccharides. In particular, it has been shown that microalgae extracts enriched in carotenoids may provide neuroprotective effects against different manifestations of AD, such as reducing oxidative stress, regulating inflammation, inhibiting A*β* accumulation, and supporting brain functions responsible for synaptic plasticity [7,8]. Moreover, specific carotenoids, such as lutein and astaxanthin, can increase neuronal survival through the Nrf2/HO-1 signaling pathway, which is crucial for cellular defense against oxidative damage and signaling pathways related to cell survival and proliferation [9,10,11]. And fucoxanthinol has been demonstrated to protect against neurodegeneration by reducing oxidative stress and inflammation in neuronal cells [12,13]. These neuroprotective effects have not yet been evaluated for other carotenoids, such as crocoxanthin, diatoxanthin, neoxanthin, and prasinoxanthin, but the antioxidant capacity of these molecules may also play a role protecting neuronal cells from oxidative damage [14].

The absorption of carotenoids in the intestine and their permeability across the BBB are also critical factors for their bioavailability and potential bioactive effects. The absorption process of carotenoids involves, respectively, their release from the food matrix by emulsification by digestive enzymes and bile salts, their dissolution in the intestinal mucosa through micelle formation, their uptake by enterocytes by passive diffusion or via carriers, and their passage into the bloodstream via the lymphatic system within chylomicrons. Finally, they are delivered to various tissues, including the brain [15,16,17]. In this regard, different studies have demonstrated that certain carotenoids, such as lutein, astaxanthin, and fucoxanthin, can cross the BBB and exert beneficial effects on neuronal health [11,13,18]. However, the knowledge on the BBB permeability and intestinal absorption of other carotenoids is still limited, but their structural similarity suggests that they may share similar absorption mechanisms [19].

*Tetraselmis chuii* is a green microalga recently approved by the EFSA as a food and dietary supplement that is being increasingly used in nutraceutical production worldwide due to its high nutritional value [20,21,22]. In addition, *T. chuii* is rich in bioactive compounds, especially carotenoids and omega-3 fatty acids [23,24,25], which makes this microalga as a promising candidate for potential neuroprotective applications. Recent studies have shown that different extracts obtained from *T. chuii* have significant antioxidant and anti-inflammatory activities [26,27]. Furthermore, in our previous study, we evaluated and compared the antioxidant, anti-inflammatory, and anti-cholinergic activities of different extracts obtained from *T. chuii* by using pressurized liquid extraction (PLE) and supercritical fluid extraction (SFE) [27]. The results demonstrated that two extracts obtained using different PLE conditions were the most promising due to their strong bioactivities, and they were chemically characterized using powerful analytical techniques (HPLC-DAD-APCI-QTOF-MS/MS and GC-QTOF-MS). As a result, it was determined that the extracts contained various carotenoids, such as neoxanthin, violaxanthin, zeaxanthin, and α- and β-carotene, with an overall higher content of these compounds in the extract obtained using 65.1% of cyclopentyl methyl ether (CPME) and 34.9% of ethyl acetate (AcOEt) at 40 °C (named OP-1) than in the extract obtained using 45.9% of CPME and 54.1% of AcOEt at 180 °C (named OP-2). However, the in vitro evaluation of the neuroprotective effects of these extracts, together with the BBB permeability and the intestinal transepithelial transport of the main carotenoids, has never been evaluated for this microalga.

One of the most used in vitro cell culture models to evaluate neuronal function, neurotoxicity, and neuroprotection is the human neuroblastoma SH-SY5Y cell line, which has been used to investigate the neuroprotective effects of carotenoids and other potential compounds against oxidative stress and neurodegenerative processes [8,28]. With regard to the in vitro models used to study the BBB permeability of natural compounds, the human brain microvascular endothelial cell line HBMEC is one of the most employed [29,30]. It provides a critical model to study the mechanisms of therapeutic agents passing into the brain and to evaluate the capacity of these compounds to affect the integrity of the BBB, which is crucial to maintaining the CNS homeostasis. Finally, the human adenocarcinoma Caco-2 cells can mimic the intestinal epithelium once differentiated, and this model is widely used to assess gastrointestinal absorption and oral bioavailability of natural and synthetic products in humans [31]. In this sense, the novelty of the present work lies in the neuroprotection evaluation of *T. chuii* extracts and the simultaneous use of both HBMEC and Caco-2 cell culture models that can provide essential information to understand the potential of specific carotenoids from the extracts to be absorbed in the gastrointestinal tract and their capacity to reach the brain.

Therefore, the main objectives of the present work were the evaluation of the neuroprotective effects of two promising bioactive extracts obtained from *T. chuii* against different insults related to AD by using a neuron-like cell culture model (SH-SY5Y), and the evaluation and comparison of the intestinal transepithelial transport and BBB permeability of the main carotenoids present in the extracts by using two well-known cell culture models (Caco-2 and HBMEC cells).

## 2. Results

### 2.1. In Vitro Neuroprotective Potential of T. chuii Extracts in SH-SY5Y Cells

**Figure 1:**
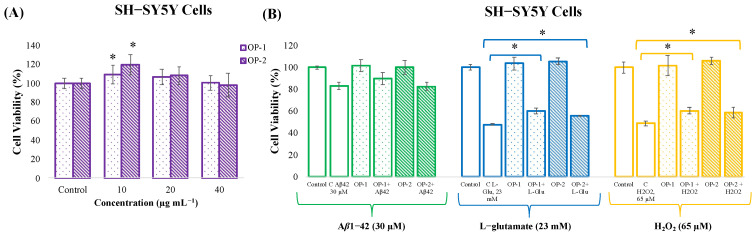
(**A**) Cell viability (%) of SH-SY5Y cells after exposure to different concentrations of *T. chuii* extracts (OP-1 and OP-2) for 24 h (* denotes statistical differences between control and extract-treated cells, *p* < 0.05). (**B**) Neuroprotective effect of *T. chuii* extracts (OP-1 and OP-2) against the neurotoxic agents A*β*1-42 (30 µM), L-glutamate (23 mM), and H_2_O_2_ (65 µM) in differentiated SH-SY5Y cells. Non-treated cells were used as control, together with only *T. chuii* extracts-treated cells at 40 µg mL^−1^. The results are expressed as the mean (*n*  =  3) ± SD. Asterisks (*) denote statistical differences between the compared groups, *p* < 0.05.

The first major objective of the present work was the evaluation of the neuroprotective effect of the *T. chuii* extracts (OP-1 and OP-2) against different insults (A*β*1-42, L-glutamate, and H_2_O_2_) by using a neuron-like cell culture model (SH-SY5Y cells). For that aim, the in vitro toxicity evaluation of OP-1 and OP-2 extracts was first assessed at different concentrations. As can be observed in Figure 1A, none of the three concentrations tested (10, 20, and 40 µg mL^−1^) significantly affected the viability of the SH-SY5Y cells after 24 h, and therefore the highest concentration (40 µg mL^−1^) was selected to evaluate its neuroprotective effects. Moreover, both OP-1 and OP-2 extracts at 10 µg mL^−1^ dose induced a significant increase in cell growth compared to the control group (*p* < 0.05).

In order to evaluate the neuroprotection capacity of OP-1 and OP-2 extracts against different insults, differentiated SH-SY5Y cells were seeded and incubated with 40 µg mL^−1^ of the extracts for 24 h. After that, the neurotoxic agents A*β*1-42, L-glutamate or H_2_O_2_ were added for another 24 h at 30 µM, 23 mM, and 65 µM concentrations, respectively. Controls containing only cell growth medium were also included to determine the maximum cell viability. As can be observed in Figure 1B, A*β*1-42 at 30 µM reduces the cell viability to 83% compared to control cells (untreated). When OP-1 or OP-2 extracts were added as a pretreatment before A*β*1-42, and although there was a slight increase up to 89% when OP-1 was used, no significant protection was observed. As for the protection against L-glutamate, it could be observed that, at 23 mM, L-glutamate decreased the cell viability to 47% compared to control cells (Figure 1B). However, when OP-1 extract was added as a pretreatment, a significant increase in the cell viability was observed (60%). To a lesser extent, a significant increase was also observed when OP-2 was added (55%), but there were no significant differences between the neuroprotective effects of both extracts. Finally, the neuroprotective effect against H_2_O_2_ was evaluated; as can be seen, at 65 µM, H_2_O_2_ decreased the cell viability to 48% compared to the control group, but when cells were pretreated with OP-1 extract, a significant increase in the cell viability was observed (60%) (Figure 1B). A similar effect was observed when OP-2 was added, as the cell viability significantly increased to 58%, but no significant differences in the cell viability were found between both extracts.

**Figure 2:**
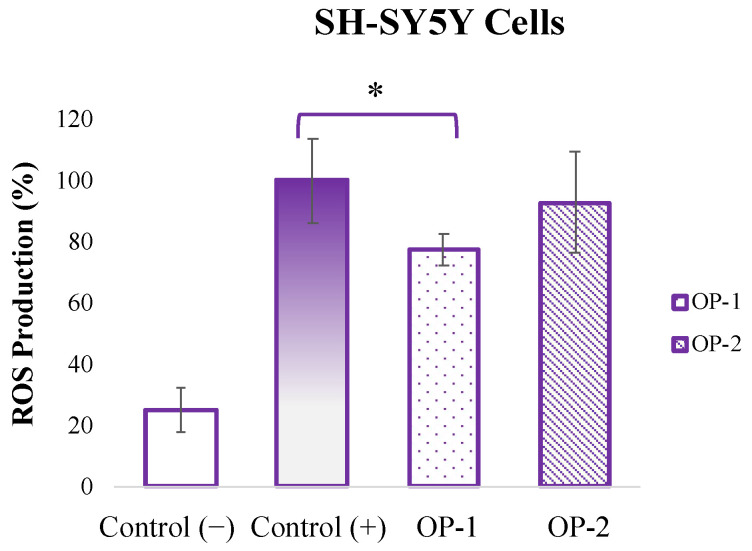
Effect of *T. chuii* extracts (OP-1 and OP-2) at 40 µg mL^−1^ on H_2_O_2_-induced ROS levels in SH-SY5Y cells. Results are expressed as the mean (*n* = 3) ± SD. Asterisks (*) denote statistical differences between the positive control (H_2_O_2_ at 65 µM) and the *T. chuii* extracts, *p* < 0.05.

Complementary to the cell viability experiments, the antioxidant capacity of OP-1 and OP-2 extracts against reactive oxygen species (ROS) production induced by H_2_O_2_ at 65 µM was evaluated. As can be observed in Figure 2, the pretreatment with OP-1 extract significantly reduced (from 100% to 77%) the H_2_O_2_-induced ROS production, while OP-2 extract only reduced it to 92% (not significant).

### 2.2. Parallel Artificial Membrane Permeability Assay for the Blood–Brain Barrier (PAMPA-BBB)

To try to obtain more insights into the BBB permeability of the carotenoids present in OP-1 and OP-2 *T. chuii* extracts, the PAMPA-BBB assay was performed. This method is commonly used in the early-stage screening of compounds. However, and after performing the UHPLC-q-TOF-MS analyses, it was determined that none of the carotenoids contained in the extracts was transported by passive diffusion through the artificial membrane, so a more complex BBB model was investigated that could help us to investigate the transport of carotenoids from *T. chuii* extracts across the brain microvascular endothelium.

### 2.3. In Vitro Toxicity and Cell Barrier Integrity Assay of T. chuii Extracts in HBMEC Cells

Since the second major objective of the present work was to investigate the transport of carotenoids from *T. chuii* extracts across the brain microvascular endothelium, without damaging the BBB, it is essential to evaluate the safety of the extracts used. Therefore, a viability assay by the MTT test was performed in HBMEC cells to determine the safe concentrations of both (OP-1 and OP-2) extracts. The tested concentrations (10, 20, and 40 µg mL^−1^) were the same used in the neuron-like cells (SH-SY5Y) and in our previous publication [27].

**Figure 3:**
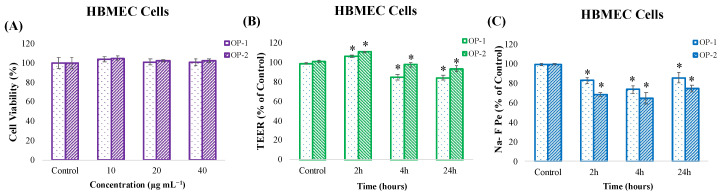
(**A**) Cell viability (%) of HBMEC cells after exposure to different concentrations of *T. chuii* extracts (OP-1 and OP-2) for 24 h. (**B**,**C**) Evaluation of the changes in the blood–brain barrier integrity of HBMEC cells after *T. chuii* extracts (OP-1 and OP-2) treatment at 40 µg mL^−1^ for different incubation times (2, 4 and 24 h) using (**B**) transendothelial electrical resistance (TEER) and (**C**) sodium fluorescein (Na-F) paracellular permeability. Asterisks (*) denote statistical differences between control and extract-treated cells at different incubation times, *p* < 0.05.

As can be observed in Figure 3A, our results demonstrate that none of the three concentrations tested significantly affected the viability of HBMEC cells.

Based on these results, the highest concentration (40 µg mL^−1^) was selected to assess the BBB integrity and transport assays. To evaluate the BBB integrity after incubation with OP-1 and OP-2 extracts at different incubation times (2, 4, and 24 h), two methods were applied: the transendothelial resistance (TEER) and the sodium fluorescein (Na-F) paracellular permeability. Results showed that applying *T. chuii* extracts for 2, 4, and 24 h in HBMEC cells largely preserved the barrier integrity, with TEER values close to or slightly above the control level (Figure 3B). These results indicate an absence of disruption of tight junctions or loss of BBB properties. In addition, the Na-F flux measurements (Figure 3C) showed that *T.chuii* extracts did not cause a significant increase in the permeability after 2, 4, and 24 h. The Na-F permeability was significantly lower than the control conditions, which could indicate a protection and improvement of the BBB functionality.

### 2.4. In Vitro Toxicity and Cell Barrier Integrity Assay of T. chuii Extracts in Caco-2 Cells

**Figure 4:**
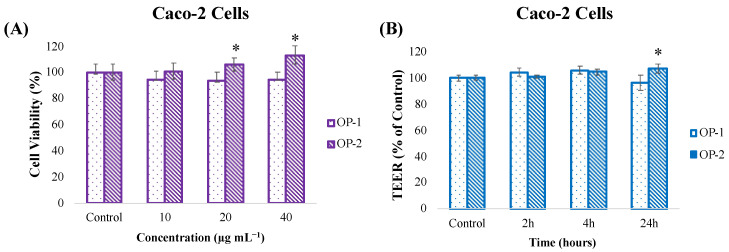
(**A**) Cell viability (%) of Caco-2 cells after exposure to different concentrations of *T. chuii* extracts (OP-1 and OP-2) for 24 h. (**B**) Evaluation of the changes in the intestinal barrier integrity of Caco-2 cells after *T. chuii* extracts (OP-1 and OP-2) treatment at 40 µg mL^−1^ for different incubation times (2, 4, and 24 h) using transendothelial resistance measurement (TEER). Asterisks (*) denote statistical differences between control and extract-treated cells at different incubation times, *p* < 0.05.

Another major objective of the present work was to investigate the transport of carotenoids from *T. chuii* extracts across the intestinal endothelium, without damaging the intestinal barrier. For that aim, a viability assay by MTT test was also performed in Caco-2 cells to determine the safe concentration of both extracts. As can be observed in Figure 4A, our results show that none of the three concentrations tested (10, 20, and 40 µg mL^−1^) was toxic to Caco-2 cells, and that OP-2 extract, in particular, significantly increased cell proliferation. Based on the results, the highest concentration (40 µg mL^−1^) was selected to assess the intestinal barrier integrity and transport assays.

Intestinal barrier integrity was evaluated by the transendothelial resistance (TEER) method after incubation of OP-1 and OP-2 extracts at different times (2, 4, and 24 h).

The results showed that the application of *T. chuii* extracts for 2, 4, and 24 h in Caco-2 cells largely preserved the intestinal barrier integrity, with TEER values close to or slightly above the control level, indicating an absence of disruption of tight junctions (Figure 4B).

### 2.5. Evaluation of Carotenoids Transport Across the BBB and the Intestinal Barrier Endothelium

Our previous study tentatively characterized 20 pigments (carotenoids and chlorophylls) in OP-1 and OP-2 *T. chuii* extracts, such as violaxanthin, zeaxanthin, lutein, crocoxanthin, fucoxanthinol, and α- and β-carotene, among others [27]. The results showed similar profiles for both extracts, with lower carotenoid content in OP-2 extract compared to OP-1. Based on these results, the obtained samples from the transport assays performed in HBMEC and Caco-2 cells were subjected to UHPLC-q-TOF-MS analysis to determine the BBB and intestinal-barrier in vitro crossing potential of the previously identified compounds. In these analyses, a peak intensity filtering threshold of 5000 (nearly four times the noise signal) was applied to obtain reliable and accurate results. After the filtering process, it was observed that the peak intensity of the compounds detected in OP-2 extract was below the threshold value. Therefore, no compound in this extract could be evaluated. The other extract, OP-1, was found suitable for evaluation after the filtering process. As a result, 14 carotenoids were identified in the upper compartment of the BBB and the intestinal barrier experiments after OP-1 extract treatment. Among them, the transport activities (Te) of six carotenoids could be calculated (Table 1), which chemical structures and extracted ion chromatograms are shown in Supplementary Figures S1 and S2.

As can be observed, fucoxanthinol was one of the carotenoids that reached the highest Te values in both cell lines. In the case of the HBMEC cell line, its permeability was 4.4% after 2 h of incubation, which increased to 18.9% after 24 h. Similarly, the calculated permeability at 2 h in Caco-2 cells (7.0%) also increased over time, reaching 18.4% after 24 h. On the other hand, crocoxanthin permeability did not show a time-dependent change in any of the cell models. However, its permeability was significantly higher in Caco-2 cells (≈12%) than in HBMEC cells (≈3%). The permeability of diatoxanthin and neoxanthin also followed a different behavior depending on the incubation time and cell model used. In the case of diatoxanthin, its permeability increased from 1.1% at 4 h to 8.1% at 24 h in the HBMEC cell model; meanwhile, it slightly increased in Caco-2 cells from 1.4% at 2 h to 4.4% at 24 h. The same happened for neoxanthin, for which the permeability increased from 1.4% at 4 h to 13.2% at 24 h in HBMEC, while it remained low (≈0.6%) in Caco-2 cells during the whole experiment. Finally, the permeability of violaxanthin and prasinoxanthin could only be quantified after 24 h of incubation in HBMEC cells, but it could not be detected in the Caco-2 cells.

Apart from the six carotenoids presented in Table 1, another seven carotenoids (diadinoxanthin, zeaxanthin/lutein, antheraxanthin, echinenone I, echinenone II, α-carotene, and β-carotene) were also detected in the upper compartment of HBMEC and Caco-2 cell transport experiments. However, they could not be detected in the lower compartment at the incubation times tested in any of the models, meaning that these carotenoids were not sufficiently transported or that the signal intensity was too low. Finally, none of the previously reported chlorophylls in OP-1 extract could be detected in the upper or lower compartments of these experiments.

## 3. Discussion

Alzheimer’s disease (AD) is a neurodegenerative disease with a multifactorial pathophysiology, including neuro-inflammation, oxidative stress, synaptic loss, and neuronal cell death. Since the interaction of multiple mechanisms plays a role in the development and progression of AD, it is essential to develop effective multi-targeted treatment strategies to prevent or slow down the progression of the disease. In this context, natural compounds, especially extracts containing components with antioxidant, anti-inflammatory, and neuroprotective properties, are attracting attention as potential therapeutic agents against AD [32].

Our previous study determined that *T. chuii* extracts obtained by PLE can inhibit acetylcholinesterase (AChE) and lipoxygenase (LOX) enzymes and exhibit potent antioxidant activity [27]. AChE inhibition may prevent cognitive function loss by protecting the cholinergic system, while LOX inhibition may help protect nerve cells by reducing neuro-inflammation. Moreover, antioxidant properties may play an essential role in slowing down the progression of AD by preventing cellular damage caused by oxidative stress. To obtain deeper knowledge of the bioactive properties of these extracts, the present study evaluated the neuroprotective effects, as well as the potential of the main bioactive compounds to affect and cross the intestinal epithelium and the BBB, by using cell culture in vitro models. However, preserving cell viability is essential to ensure that the extracts can be safely applied without damaging the cells they are intended to protect. Toxicity results of the present work showed that *T. chuii* extracts at 40 µg mL^−1^ maintained (or even increased) the cell viability in three different cell culture models (SH-SY5Y, HBMEC, and Caco-2), which is in good agreement with our previous results obtained from other cell culture models [27]. Furthermore, OP-2 extract slightly increased cell proliferation with respect to OP-1 extract, which might be a consequence of their different composition (OP-1 exhibited high concentrations of phospholipids and prenol lipids, while OP-2 contained more glycerolipids and sphingolipids). Previous studies have also shown that microalgae extracts can positively affect cell growth. For instance, fucoxanthin, lutein, and astaxanthin obtained from various microalgae promoted cell viability and proliferation rates at specific concentrations (100–500 µg mL^−1^) [33,34]. In contrast, other works have shown toxic effects of microalgae extracts above 20 µg mL^−1^ [8]. Therefore, toxicity tests using cell culture models are essential to determine the safe and effective doses of each specific extract.

In the pathophysiology of neurodegenerative diseases, oxidative stress and damage to cells caused by neurotoxic agents play a critical role in the progression of the disease [35]. For example, accumulation of A*β* plaques, one of the characteristic pathological features of AD, lead to neuronal toxicity, causing cell death, synaptic dysfunction, and damage to brain tissue. In addition, excessive glutamate accumulation leads to excitotoxicity, creating a toxic effect on neurons. Moreover, H_2_O_2_, an important trigger of oxidative stress in cells, increases the production of ROS, leading to damage to cellular structures and cell death [36,37]. In this context, the neuroblastoma SH-SY5Y cell model has been widely used as an in vitro model for examining, for instance, the activity of different natural compounds as A*β*1-42 inhibitors [28]. Making use of the same cell model, the present study evaluated the neuroprotective potential of pretreating SH-SY5Y cells with *T. chuii* extracts (at a non-toxic concentration) against the toxic effects of A*β*1-42, L-glutamate, and H_2_O_2_. The results showed that the pretreatment with 40 µg mL^−1^ of *T. chuii* extracts could partially reduce cell death caused by the neurotoxic agents L-glutamate and H_2_O_2_, but its effect against 30 µM A*β*1-42 was limited. These effects were higher for OP-1 extract than for OP-2, which might be a consequence of a higher carotenoid content in OP-1. In line with this, previous studies have shown that different carotenoids can protect the cellular components against ROS, enhance the endogenous antioxidant systems, and modulate neuroinflammation-related mechanisms [38,39]. In addition, it has been shown than β-carotene (higher in OP-1 extract than in OP-2) can alter the aggregation pathway of Aβ, but it does not completely inhibit the fibril formation [40], which could explain the slight but not significant protection observed for OP-1 against A*β*1-42 insult. Other possible mechanisms might be the regulation of different transcription factors involved in the expression of detoxification and antioxidant response genes [41], or the prevention of mitochondrial impairment [42]. In this last study, astaxanthin promoted mitochondria-related cytoprotection in SH-SY5Y cells exposed to L-glutamate by a mechanism dependent on the Nrf2/HO-1 axis. Moreover, lutein (higher in OP-1 extract than in OP-2) was also shown to provide protection for SH-SY5Y neuroblastoma cells against L-glutamate-induced oxidative stress and proinflammatory cytokine production [43], which could explain the higher protection of OP-1 extract (up to 60%) than OP-2 extract (up to 55%) against L-glutamate insult. Other studies have also confirmed the neuroprotective effect of carotenoid enriched microalgae extracts against H_2_O_2_, A*β*1-42, and L-glutamate. For example, it was determined that a *Chlorella variabilis* extract significantly reduced H_2_O_2_-induced cytotoxicity by providing cell viability up to 73.7% at 75 µg mL^−1^ [44]. Furthermore, it was reported that a pretreatment with a *Dunaliella salina* extract reduced the loss of cell viability caused by A*β*1-42 by 15% and alleviated L-glutamate-induced oxidative stress by approximately 10% [8]. And more recently, the same *D. salina* extract showed neuroprotective effects by preventing the A*β*-peptide-induced paralysis in a *Caenorhabditis elegans* AD model when applied at a dose of 50 μg mL^−1^ [7]. The present study also evaluated the level of intracellular ROS production, a direct indicator of the oxidative stress, after H_2_O_2_ stimulation. As a result, it was found that *T. chuii* OP-1 extract effectively reduced ROS production. This protective effect might be related to the electron-donating abilities of carotenoids that can prevent lipid peroxidation in neuronal cells and reduce cellular oxidative damage by scavenging free radicals and ROS, and preserving neuronal integrity [14,45]. The study by González-Peña et al. (2021) supports this, showing that carotenoids can inhibit lipid peroxidation [46]. Carotenoids may also exert their antioxidant effects through indirect pathways mediated by gene expression related to ROS scavenging and antioxidant defenses. For example, studies by Shen et al. (2017) indicated that carotenoids can induce the expression of genes responsive to oxidative stress and increase overall antioxidant capacity [47]. They have also been shown to facilitate cellular signaling that can reduce oxidative stress-induced apoptosis [48]. Moreover, it has been reported that carotenoids can exhibit synergistic effects when used together, enhancing their beneficial properties [49]. For example, Shi et al. (2004) showed that lycopene and other carotenoids provided stronger protection against oxidative damage when applied as mixtures [50]; and synergistic effects to modulate oxidative stress and cell death progression were observed between astaxanthin, β-carotene, and lutein [51]. Finally, it has also been demonstrated that carotenoids exhibit synergistic effects with other bioactive molecules, such as flavonoids [52].

Next, in vitro BBB permeability of the OP-1 and OP-2 compounds was studied using the PAMPA-BBB assay [53,54]. The BBB is a semi-permeable structure that acts as a boundary between the CNS and the bloodstream. This PAMPA test allows for studying the passive diffusion of molecules through the BBB, a critical factor in drug development and nutritional bioavailability. However, the results obtained by UHPLC-q-TOF-MS after the PAMPA-BBB assay determined that none of the carotenoids was transported through this model. These results might be due to the physicochemical properties of carotenoids and the structural features of the PAMPA-BBB model. For instance, carotenoids are lipophilic compounds that generally show poor solubility in aqueous media, thus posing a challenge for their absorption and subsequent transport across biological membranes [8,55]. Unlike small and lipophilic molecules that can diffuse passively, carotenoids may require specific transport proteins or carriers to facilitate their movement across the barrier; and the PAMPA-BBB assay uses a lipid membrane that does not fully mimic these complex interactions and transport mechanisms [56]. As a result, the PAMPA-BBB model does not accurately reflect the permeability of carotenoids [57], as we confirmed the presence of the carotenoids in the PAMPA-BBB solution before the experiment.

The membrane permeability of carotenoids was also evaluated in vitro using two cell culture models (HBMEC and Caco-2) that mimic the BBB and the intestinal epithelium. The BBB consists of brain microvascular endothelial cells (BMECs) and supporting cells, such as astrocytes, pericytes, and a basement membrane [58]. Thanks to the tight junctions between its cells, the BBB’s selective permeability allows the transport of oxygen, glucose, and other nutrients needed by the brain, while preventing the passage of harmful substances. This makes it difficult for many drugs to reach the brain and causes difficulties in the treatment of neurological diseases [58]. Therefore, examining the bioavailability of neuroprotective and neuroactive compounds, and their ability to cross the BBB, plays a critical role in understanding the potential therapeutic effects of these compounds. Together with these assays, the TEER measurement is used to evaluate the tight junctions and barrier integrity between cells. A decrease in TEER values indicates that the tight junctions are disrupted and the barrier structure is damaged. Complementary to this measurement, the Na-F permeability analysis indicates changes in the function of the barrier. Accumulation of Na-F in the lower compartment indicates that the barrier structure is disrupted and, as a result, the barrier functionality is reduced. Based on these assumptions, the BBB integrity was evaluated by measuring TEER and Na-F flux (Figure 3). Results determined that *T. chuii* extracts largely preserved the barrier integrity after 2, 4, and 24 h of application in the cell-based in vitro BBB model, as the TEER values were close to the control level, and the Na-F flux measurements did not show a significant increase in permeability, thus confirming that the membrane integrity was not disrupted and there was no uncontrolled passage of compounds. The observed effects are not specific or exclusive for carotenoid compounds, and they have to be experimentally tested in each particular study. For instance, similar effects were noted in previous studies, where extracts containing different classes of compounds, such as triterpenoids and caffeic acids obtained from *Olea europaea*, or monoterpenoids from *Lavandula officinalis*, were applied to the same BBB model [30]. In another study, it was determined that some sesquiterpene lactones obtained from chicory increased barrier tightness and provided a significant increase in TEER values, which partly depended on their chemical structure [59].

To highlight the potential connection between the BBB and the intestinal epithelium, it is important to note that both barriers are epithelial cell-based structures and share certain common features. Both the BBB and the intestinal barrier provide selective permeability using cell-to-cell connections and carrier proteins. These common features can help in predicting the behavior of bioactive compounds and their potential to cross the BBB in permeability studies of the intestinal barrier [60]. A compound’s ability to easily cross the intestinal barrier also indicates that it has the potential to travel through the body, cross the BBB, and reach the brain. However, the BBB is much more selective due to its tighter junctions and more specific transport mechanisms. Therefore, the results obtained in the intestine only play a supporting and complementary role in predicting BBB permeability [61]. In the present work, it was determined that the barrier integrity of Caco-2 cells was also largely preserved after 2, 4, and 24 h of treatment with *T. chuii* extracts at a concentration of 40 μg mL⁻¹, as the TEER values were close to or slightly above the control level (Figure 4). Previous studies performed on the same Caco-2 model have shown that other compounds, such as rhamnogalacturonan, can increase the TEER by improving intestinal barrier function [62]. Moreover, kaempferol has been found to enhance the tight junction barrier integrity, while quercetin was found to strengthen TEER by increasing claudin-4 expression in a dose-dependent manner [63]. Some peptides can also improve the tight junction barrier by increasing occludin expression [64].

Finally, the transport efficiency of carotenoids from *T. chuii* extracts was evaluated in vitro in HBMEC and Caco-2 cell culture models. According to the UHPLC-q-TOF-MS analyses, only OP-1 extract was found suitable for this evaluation. Overall, the results obtained (Table 1) highlight that the transport efficiency of the carotenoids present in *T. chuii* extract depend on their abundancy and their chemical structure, as well as the cell model used and the incubation time. All of these parameters are essential for evaluating the potential of carotenoids to reach the CNS and exert their beneficial effects. For instance, fucoxanthinol was the compound with the highest and similar permeability in both cell lines (more than 18% after 24 h of incubation), while crocoxanthin was highly absorbed from the intestinal epithelium (12.1%), and its potential to cross BBB was low (3%). Conversely, the BBB permeability of diatoxanthin and neoxanthin increased over time up to 8.1% and 13.2%, respectively, while the maximum in Caco-2 cells remained at 4.4% (for diatoxanthin) and 0.6% (for neoxanthin). Finally, violaxanthin and prasinoxanthin showed permeability only in the BBB model and could not pass through the intestinal epithelium. The differences observed for these compounds could be due to sample degradation or compound instability (although we could detect these compounds in the upper compartment of Caco-2 experiments), or because Caco-2 cells could efficiently absorb and metabolize these compounds without releasing them into the basolateral compartment, but future studies are needed to verify this hypothesis. These results are in good agreement with other studies that have investigated the absorption and bioavailability of carotenoids in the gastrointestinal tract using the Caco-2 model. For example, carotenoids such as canthaxanthin, crocoxanthin, α-carotene, cryptoxanthin, violaxanthin, lutein, and zeaxanthin have been reported to exhibit different permeability levels [55]; and it is known that this process is also affected by micelle formation, a process mainly promoted by unsaturated fatty acids [65]. Furthermore, carotenoids that can cross the BBB have therapeutic potential in preventing oxidative stress and inflammation in AD and other neurodegenerative disorders [66]. For instance, astaxanthin can cross the BBB due to its favorable interactions with the lipid bilayer, and it has exhibited strong neuroprotective effects [67,68]. Similarly, lycopene has demonstrated both BBB permeability and neuroprotective properties [69]. Additionally, fucoxanthinol has been reported to cross the BBB, and it may protect against neurodegenerative diseases [12,70]; and neoxanthin may exhibit a comparable potential for crossing the BBB due to its structural similarity to fucoxanthin [71]. Still, the specific BBB transport mechanisms of carotenoids have not yet been fully elucidated, but it is thought that carriers such as lipoproteins may help these molecules reach the brain [72]. In this regard, different approaches are being explored to increase the naturally limited permeability of these molecules. For example, nanotechnological approaches can facilitate the passage of biological barriers by increasing the solubility and stability of carotenoids [73]. Many approaches include the use of biopolymeric and lipid-based nanocarriers, which can be found in nature and are cheap, non-toxic, and non-reactogenic [74]. Biopolymer-based nanocarriers include polysaccharides (chitosan, alginate, starches, cellulose, pectin, and gums), proteins (whey protein, caseins, gelatin, soy proteins, cereal proteins, and pulse proteins), or nano-hydrogels, while monoglycerides, triglycerides, phospholipids, and cholesterol are used as lipid-based nanocarriers. These strategies also increase the protection of carotenoids during processing, storage, and digestion processes, but other nanoparticles are being designed as sensitive delivery systems, releasing their cargo only in the required areas depending on pH, temperature, or enzyme activity [75]. Moreover, targeting carrier proteins such as organic anion transporting polypeptides (OATPs) can enable carotenoids to effectively cross the BBB by taking advantage of their lipophilic properties [76]. Ensuring that these compounds effectively reach the brain through these innovative and targeted strategies is a critical requirement to harness the potential neurological benefits of carotenoids truly.

## 4. Materials and Methods

### 4.1. Samples, Chemicals, and Reagents

*Tetraselmis chuii* microalga was kindly donated by Fitoplancton Marino S.L. (El Puerto de Santa María, Cádiz Spain). *T. chuii* was dried and milled, and the powder was vacuum-packed (C400 Multivac. Wolfertschwenden, Germany) and stored at −18 °C.

SH-SY5Y, HBMEC, and Caco-2 cells were obtained from ATCC^®^ (Rockville, MD, USA). Roswell Park Memorial Institute (RPMI) 1640 medium, fetal bovine serum (FBS), non-essential amino acids (NEAAs), minimal essential medium (MEM) vitamins, sodium pyruvate, L-glutamine, trypsin–EDTA, antibiotic solution (including penicillin and streptomycin), thiazolyl blue tetrazolium bromide (MTT), PBS, dichlorodihydrofluorescein diacetate (DCF-DA), n-dodecane, porcine polar brain lipid (PBL), PAMPA-BBB 96 well-donor plate (Cat. MAIPNTR10), PAMPA-BBB 96 well-acceptor plate (Cat. MATRNPS50), and sodium fluorescein (molecular weight, 376 Da) were purchased from Sigma-Aldrich (St. Louis, MO, USA). NuSerum IV was obtained from Corning Costar Corp (New York, NY, USA). HPLC-grade solvents such as ethanol (EtOH), isopropanol, methyl tert-butyl ether (MTBE), methanol, CPME, AcOEt, hydrogen peroxide (H_2_O_2_), and dimethyl sulfoxide (DMSO) were obtained from VWR Chemicals (Barcelona, Spain). Ultrapure water was obtained from a Millipore system (Billerica, MA, USA). Sea sand for PLE extractions was obtained from Panreac Quimica (Barcelona, Spain). Transwell cell culture chambers (12-well plates) with 3.0 μm pore size and rat tail collagen type I solution (CLS354236) were purchased from Corning Life Sciences (Tewksbury, MA, USA). Brain-derived neurotrophic factor (BDNF) was obtained from Hello Bio (Dunshaughlin, Republic of Ireland). Light microscopy for monitoring cell cultures was performed using a Nikon Inverted Microscope (ECLIPSE, TE2000-U, Tokyo, Japan). A Universal Microplate Analyzer, models AOPUS01 and AI53601 (Packard BioScience, Meriden, CT, USA), and 96-well plates were all purchased from Costar, Corning, NY, USA. Hank’s balanced salt solution (HBSS) and Dulbecco’s Modified Eagle’s Medium (DMEM) with L-glutamine and 4.5 g L^−1^ glucose were obtained from Lonza (Basel, Switzerland). Krebs–Ringer solution (HEPES-buffered) was purchased from Thermo Scientific (Waltham, MA, USA).

### 4.2. Carotenoids-Enriched Microalga Extract

In this study, *T. chuii* OP-1 and OP-2 extracts were obtained using a Dionex accelerated solvent extractor (ASE 200, Dionex Corporation, Sunnyvale, CA, USA), according to our previous work [27]. Briefly, 1.0 g of dried microalga biomass was placed into an 11 mL stainless-steel extraction cell, and it was sandwiched between two layers of sea sand (2.0 g each). Then, the extraction was performed for 20 min at 10.3 Mpa (for 1 cycle) by using a mixture of CPME (65.1%) and AcOEt (34.9%) at 40 °C to obtain OP-1 extract, and a mixture of CPME (45.9%) and AcOEt (54.1%) at 180 °C to obtain OP-2 extract. These extracts were extensively characterized in our previous article by GC-QTOF-MS and HPLC-DAD-APCI-QTOF-MS/MS [27].

### 4.3. Cell Culture Assays in SH-SY5Y Cells

#### 4.3.1. Toxicity Evaluation of *T. chuii* Extracts

SH-SY5Y neuroblastoma cells were cultured in the same conditions as previously described [8]. Cells were grown in DMEM/F12 medium supplemented with 10% FBS, 100 U mL^−1^ penicillin, 100 µg mL^−1^ streptomycin, and 250 ng mL^−1^ antimycotic in a 95% humidified atmosphere with 5% CO_2_ at 37 °C. Cell culture medium was changed every three days to ensure optimal growing conditions. The differentiation process began when cells were transferred to a fresh medium containing 10% FBS (day 0 of differentiation). After 24 h (day 1), the cell culture medium was replaced with fresh medium containing 1% FBS and 10 µM retinoic acid (RA). After 3 days (day 4), the cell culture medium was replaced with fresh medium containing 1% FBS, 10 µM RA, and 50 ng mL^−1^ BDNF. On day 7, differentiated cells were trypsinized and seeded in 24-well plates at a density of 42,000 cells/cm². At the end of the 24 h incubation period, the differentiation of SH-SY5Y cells into neuron-like phenotype was confirmed morphologically, the *T. chuii* extracts (OP-1 and OP-2) were added at different concentrations, and the cells were incubated for another 24 h.

Then, the viability was determined by the MTT assay as below. At the end of the 24 h incubation period, the medium was removed, and 0.5 mg mL^−1^ MTT solution was added to each well. The cells were incubated at 37 °C for 3 h. At the end of the incubation, MTT was carefully removed, and formazan crystals in the wells were dissolved by adding 100 µL of DMSO. The absorbance of the solution was measured by a plate reader at 570 nm wavelength. Cell viability was calculated by comparing the absorbance values of treated cells with the control group (ethanol-treated cells). Ethanol concentration did not exceed 0.4% (*v*/*v*). All the experiments were performed in triplicate.

#### 4.3.2. Neuroprotection Evaluation of *T. chuii* Extracts

Three different experiments were performed to evaluate the neuroprotection capacity of OP-1 and OP-2 *T. chuii* extracts: neuroprotection against A*β*1-42, L-glutamate, and H_2_O_2_ as in [8]. Briefly, SH-SY5Y cells were plated as described above, and 24 h after cell attachment, cells were pretreated with non-toxic concentrations of OP-1 or OP-2 extracts (40 μg mL^−1^) for 24 h. In the control group, DMEM/F12 cell medium supplemented with 1% FBS, 100 U mL^−1^ penicillin, 100 μg mL^−1^ streptomycin, 250 ng mL^−1^ antimycotic, and 0.4% ethanol was used. The next day, cells were incubated with either cell medium alone (control), A*β*1-42 (30 μM), L-glutamate (23 mM), and H_2_O_2_ (65 μM) for 24 h. Cell viability was assessed by MTT assay at the end of the experiments. All the experiments were repeated three times.

#### 4.3.3. Antioxidant Capacity of *T. chuii* Extracts

The antioxidant capacity of *T. chuii* extracts (OP-1 and OP-2) against oxidative stress induced by H_2_O_2_ was determined following the method described by Lin et al. (2000), with slights modifications [77]. Briefly, differentiated SH-SY5Y cells were seeded at a density of 5 × 10^3^ cells/well in 96-well plates, and 24 h after cell attachment, the cells were pretreated with non-toxic concentrations of OP-1 and OP-2 extracts (40 μg mL^−1^) for 24 h. The next day, the medium in the wells was removed, and 100 μL of DCFH-DA (50 μM, dissolved in DMEM/F12 without FBS) was added to each well. The cells were incubated at 37 °C for 1 h. Then, cells were washed with PBS, and 100 μL of H_2_O_2_ (65 μM) was added to each well. Fluorescence measurement was performed in a Synergy HT plate reader (BioTek Instruments Inc., Winooski, VT, USA) at 485 nm excitation and 530 nm emission wavelengths for 1.5 h at 37 °C. All the experiments were repeated three times.

### 4.4. Parallel Artificial Membrane Permeability for the Blood–Brain Barrier (PAMPA-BBB)

To evaluate the BBB permeability of the bioactive compounds present in OP-1 and OP-2 extracts, the parallel artificial membrane permeability test (PAMPA-BBB), developed by Di et al. (2003), was applied [53]. Briefly, BBB solution was prepared by dissolving 8 mg of PBL and 4 mg of cholesterol in 600 μL of n-dodecane, and *T. chuii* extracts (OP-1 and OP-2) were dissolved in PBS (5 mM pH 7.4) as a starting solution at 500 μg mL^−1^ (EtOH/buffer, 1:1, *v*/*v*). Then, 5 μL of BBB solution was adsorbed onto the filter membrane of the donor 96-well plate, and the acceptor 96-well plate was filled with 350 μL of buffer. Then, 200 μL of the starting solution containing extract was added to the donor microplate, which was mounted like a sandwich on the acceptor microplate, and incubated at 37 °C in the dark for 4 h with agitation. After the incubation, 200 μL of each donor and acceptor plate were dried, and each sample was re-dissolved in 50 μL of EtOH. These samples were injected into the UHPLC-q-TOF-MS system to try to identify and compare the bioactive compounds present in both the donor and the acceptor solutions.

### 4.5. Cell Culture Assays in HBMEC Cells

#### 4.5.1. Toxicity Evaluation of *T. chuii* Extracts

The HBMEC cell line was cultured using the same protocol as in Sánchez-Martínez et al. (2023) [30]. Briefly, cells were cultured in RPMI 1640 medium containing 10% fetal bovine serum (FBS), 10% NuSerum IV, 1% non-essential amino acids (NEAA), 1% minimum essential medium (MEM), 1 mM sodium pyruvate, 2 mM L-glutamine, and 1% antibiotic–antimycotic solution. Cells were maintained at 37 °C in a humidified atmosphere containing 5% CO_2_ and were used in the experiments between passages 5 and 10. For toxicity tests, HBMEC cells were seeded in a volume of 200 µL and at a density of 2.5 × 10^4^ cells mL^−1^ into wells coated with rat tail collagen-I (100 μg mL^−1^) using 96-well plates. After 24 h, different concentrations of *T. chuii* extracts (OP-1 and OP-2) were added, and the cells were incubated for another 24 h. Then, the cell viability was determined by the MTT test, as described above. All the experiments were performed in triplicates.

#### 4.5.2. Blood–Brain Barrier Transport Study of *T. chuii* Extracts

The HBMEC cell line was used as a simplified in vitro model of the BBB endothelium. For this, cells were seeded at a density of 1.6 × 10^5^ cells/cm^2^ into 12-well Transwell^®^ plates (0.4 μm pore, Corning Costar Corp., Corning, NY, USA) coated with rat tail collagen-I (100 μg mL^−1^) [30]. Medium was changed every second day until a monolayer was formed (10 days), and the transport studies were initiated.

During transport studies, the medium in the upper chamber was removed, and 0.5 mL of non-toxic concentrations of OP-1 or OP-2 extracts (40 μg mL^−1^) was added (in duplicates). Medium containing 0.4% ethanol was used as the control group, and the lower chamber was filled with 1.5 mL of medium. The cells were incubated under these conditions for 2, 4, and 24 h. After incubation time, cell culture medium from both the upper and lower compartments was collected, dried using a freeze dryer (Martin Christ, Osterode am Harz, Germany), and stored at −80 °C until the chemical characterization was performed to assess the transport efficiency of the bioactive compounds.

The transport efficiency of bioactive compounds across the cell layer (Te) was calculated using the Equation (1) proposed by Pogačnik et al. (2016) [78]:

Te = C_Lower_/C_Upper_ × 100% (1)

This calculation was performed to understand whether the compound crosses the cell layer and how efficiently it is transported. Here, C_Lower_ represents the relative concentration (peak area) of the compound in the lower compartment, and C_Upper_ represents the relative concentration (peak area) of the compound in the upper compartment.

#### 4.5.3. Cell Barrier Integrity

Transendothelial electrical resistance (TEER) and sodium fluorescein (Na-F) paracellular permeability are two commonly used parameters to measure cell integrity [30]. These parameters were used to evaluate the effects of each extract on the BBB epithelium to prevent false-positive results of natural compounds in the lower compartment in case of disruption of the cell layer.

##### Transendothelial Electrical Resistance (TEER)

Transendothelial electrical resistance (TEER) measurements were performed in duplicate, as previously described [30]. These measurements were used to determine the electrical resistance of the endothelial cell monolayer and were evaluated using the EndOhm™ chamber connected to the EVOMX resistance meter (World Precision Instruments, Inc., Sarasota, FL, USA). TEER readings were taken immediately before (0 h) and at 2, 4, and 24 h after the addition of the extracts. After subtracting the resistance values of the blank inserts (without cells and extracts), these values were multiplied by the surface area of the inserts (1.12 cm²), and the results were expressed in Ω × cm². Then, the TEER values were compared with the mean values of the control group (without extracts) to determine the percent change in resistance. After the final TEER measurements were taken, the medium from both compartments (upper and lower) was collected and used for the transport studies described below.

##### Sodium Fluorescein (Na-F) Paracellular Permeability

The Na-F (sodium fluorescein) permeability test was performed as previously described [30]. This test was used to measure the paracellular permeability of the junctions between cells after the influence of the extracts. Once the TEER measurements and the collection of the cell culture medium were performed, the inserts containing the cells were washed twice with Ringer–HEPES buffer. These inserts were then placed in new 12-well plates containing 1.5 mL of Ringer–HEPES buffer, and 0.5 mL of 10 μg mL^−1^ Na-F dissolved in the same buffer was added to the upper compartments to be in contact with the cells. The inserts were transferred to new wells containing Ringer–HEPES solution after incubation at 37 °C for 20, 40, and 60 min, protected from light. At the end of the incubation periods, the inserts were removed, and the Na-F concentration in the lower chamber was analyzed using a fluorescence spectrophotometer (Cytation 5, BioTek Instruments, Winooski, VT, USA; excitation, 460 nm; and emission, 515 nm). The Na-F flux through the cell layers without extracts (control) and through the cell-free inserts (blanks) was also measured. This was performed to determine the true effect of the extracts and to avoid false-positive results.

Finally, the transendothelial permeability coefficient (Pe) was calculated as previously described by Deli et al. (2005) [79]. This coefficient quantifies how much Na-F is transported across the cell layer and the permeability of the cell layer. Briefly, the clearance volume of Na-F permeation into the lower compartment was determined using Equation (2):

Clearance (μL) = C_Lower_ × V_Lower_ × C_Upper_^−1^ (2)

where C_Lower_ and C_upper_ represent the Na-F concentration in the lower and upper compartment, respectively; and V_Lower_ represents the volume of the lower compartment (1.5 mL). Then, the average volume cleared was plotted versus time to obtain the clearance slopes, and the permeability of the endothelial monolayer (PS_e_) was calculated using a linear regression, considering the clearance slopes after the extract treatments (PS_time−plotted_) and the blank inserts without cells (PS_insert_), following Equation (3):

PS_e_^−1^ = PS_time-plotted_^−1^ − PS_insert_^−1^ (3)

PS_e_ divided by the surface area (1.12 cm^2^) generates the endothelial permeability coefficient [Pe (cm/s)]. This value was compared to the control group without the addition of extracts, expressing the change in permeability of the cell layer as a percentage.

### 4.6. Cell Culture Assays in Caco-2 Cells

#### 4.6.1. Toxicity Evaluation of *T. chuii* Extracts

Caco-2 cells were grown in high-glucose DMEM supplemented with 10% fetal bovine serum, 2 mM L-glutamine, 1% non-essential amino acid solution, and 1% penicillin-streptomycin solution in a humidified atmosphere (5% CO_2_, 37 °C). Cells from passages 10–20 were used for all experiments. For viability tests, Caco-2 cells were seeded at a density of 2 × 10^4^ cells mL^−1^ using 96-well plates. After 24 h, different concentrations of *T. chuii* extracts (OP-1 and OP-2) were added, and the cells were incubated for another 24 h. Cell viability was determined in three replicates by the MTT assay, as described above.

#### 4.6.2. Intestinal Transepithelial Transport Study of *T. chuii* Extracts

Caco-2 cells were grown according to the protocol described by Rojo-Poveda et al. (2020) to evaluate the intestinal permeability [80]. Briefly, Caco-2 cells were seeded into inserts (0.4 μm pore, Corning Costar Corp., Corning, NY, USA) in 12-well plates. Then, 0.5 mL of complete DMEM culture medium containing approximately 300,000 cells was applied to the apical surface (AP) of each insert, and 1.5 mL of the same culture medium was added to the basolateral side (BL). Cells were allowed to grow for 21 days to differentiate and form a monolayer, changing the culture medium every 2–3 days.

The Caco-2 transport studies were performed using the same procedures as for HBMEC experiments (described in Section 4.5.2 and Section 4.5.3), but using DMEM as cell culture medium and recording TEER measurements.

### 4.7. Quantification of Carotenoids in the Transport Assays

#### 4.7.1. Carotenoid Extraction

Obtained samples from the transport assays performed in HBMEC and Caco-2 cells were extracted to recover and concentrate the bioactive compounds in the upper and lower compartments and to prevent possible interferences of the cell culture medium in the analyses. Briefly, dried samples were resuspended in 0.5 mL of H_2_O and mixed with vortex for 10 s. Then, 0.5 mL of AcOEt was added, mixed with vortex for 10 s, and centrifuged at 14,000 rpm for 5 min. After centrifugation, the supernatant (AcOEt) was carefully transferred to a 1.5 mL Eppendorf tube and dried in a SpeedVac (SPD1030, Thermo Fisher Scientific) device at 40 °C and 13 mbar pressure. Finally, the dried extracts were resuspended in 25 μL of pure ethanol for their subsequent analysis.

#### 4.7.2. Liquid Chromatography–Tandem Mass Spectrometry (UHPLC-Q-TOF-MS)

The analysis of the obtained samples from HBMEC cells, Caco-2 cells, and PAMPA-BBB experiments was carried out using the same conditions and instrumentation as in our previous study [27]. Briefly, 10 μL of each sample was injected into an Agilent 1290 UHPLC system equipped with a DAD detector and coupled to an Agilent 6540 q-TOF/MS equipped with an orthogonal atmospheric pressure chemical ionization (APCI) source, all from Agilent Technologies (Santa Clara, CA, USA). Carotenoids were separated using a Thermo Fisher Scientific Accucore C30 column (2.6 μm, 4.6 × 50 mm), applying a flow rate of 0.8 mL min^−1^ at 30 °C. Methanol/MTBE/water (90:7:3%) and methanol/MTBE (10:90%) were used as mobile phases, and the chromatographic gradient started with 0%B, changing it to 100%B within 12 min. This was kept constant for 1.5 min before returning to the initial conditions.

The results were processed using the Agilent Mass Hunter Qualitative software (version B.10.0). A peak intensity threshold of 5000 (nearly 4 times the noise signal) in the upper compartments was applied to obtain reliable and accurate results, and a relative quantification was performed. Peaks below this threshold value were excluded from the analysis.

### 4.8. Statistical Analysis

For statistical data analysis, T-test and one-way ANOVA, followed by Tukey HSD test, were performed. Statistically significant differences were considered when *p* < 0.05.

## 5. Conclusions

The present study comprehensively evaluated the neuroprotective effects of *T. chuii* extracts obtained by green extraction techniques and, for the first time, carried out the evaluation and comparison of the intestinal transepithelial transport and BBB permeability of the main carotenoids present in the extracts by using two well-known cell culture models. The findings revealed that *T. chuii* extracts exhibited significant neuroprotective effects in a neuron-like cell model exposed to neurotoxic agents, such as L-glutamate and H_2_O_2_, as well as a partial antioxidant capacity against ROS production induced by H_2_O_2_. The experiments performed using the PAMPA-BBB model clearly indicated that this test does not fully reflect the transport potential of these molecules. On the other hand, the cell-based BBB and the intestinal transport results revealed that these extracts do not alter the integrity of the cell barriers and that different carotenoids (such as fucoxanthinol, crocoxanthin, diatoxanthin, neoxanthin, violaxanthin, and prasinoxanthin) have diverse permeabilities depending on the incubation time and the cell model used, with fucoxanthinol being the compound that exhibited the highest permeability. In conclusion, the study’s findings show that *T. chuii* extracts and the carotenoids that they contain have neuroprotective potential, but further studies are needed to increase their absorption and to evaluate their transport mechanisms and the cellular processes involved.

## Figures and Tables

**Table 1:** Carotenoid transport efficiency (Te) values of *T.chuii* extracts in HBMEC and Caco-2 cell lines after 2, 4, and 24 h of incubation. SEM: standard error of the mean.

Compound	Molecular Formula	Molecular Weight (*m*/*z*)	Te% ± SEM					
			HBMEC Cells			Caco-2 Cells		
			2 h	4 h	24 h	2 h	4 h	24 h
Fucoxanthinol	C_40_H_56_O_5_	617.4191	4.4 ± 1.1 ^c^	5.1 ± 0.1 ^b^	18.9 ± 2.3 ^a^	7.0 ± 0.2 ^c^	8.6 ± 2.2 ^b^	18.4 ± 7.2 ^a^
Diatoxanthin	C_40_H_54_O_2_	567.4208	n.d	1.1 ± 0.1 ^b^	8.1 ± 3.4 ^a^	1.4 ± 0.1 ^c^	2.3 ± 0.3 ^b^	4.4 ± 0.9 ^a^
Neoxanthin	C_40_H_56_O_4_	601.4266	n.d	1.4 ± 0.2 ^b^	13.2 ± 4.3 ^a^	0.5 ± 0.3 ^b^	0.4 ± 0.1 ^b^	0.6 ± 0.4 ^a^
Violaxanthin	C_40_H_56_O_4_	601.4258	n.d	n.d	13.9 ± 6.0	n.d	n.d	n.d
Prasinoxanthin	C_40_H_56_O_4_	601.4235	n.d	n.d	8.9 ± 2.3	n.d	n.d	n.d
Crocoxanthin	C_40_H_54_O	551.4229	3.4 ± 3.2 ^a^	0.9 ± 0.3 ^b^	2.7 ± 0.6 ^a^	12.0 ± 9.5 ^a^	11.8 ± 5.6 ^a^	12.1 ± 1.5 ^a^

Different superscript letters (a, b, c) in the same row indicate significant differences between samples after ANOVA with Tukey’s post hoc test, *p*-value < 0.05.

## Data Availability

Data are contained within the article.

## Supplementary Materials

The following supporting information can be downloaded at https://www.mdpi.com/article/10.3390/ph18050629/s1. Figure S1: Chemical structures of quantified carotenoids; Figure S2: Extracted ion chromatograms (EICs) of quantified carotenoids in HBMEC and Caco-2 cells, after different incubation times (2 h, 4 h, and 24 h).

## References

[1] Lamisa A.B., Ahammad I., Bhattacharjee A., Hossain M.U., Ishtiaque A., Chowdhury Z.M., Das K.C., Salimullah M., Keya C.A. (2024). A Meta-Analysis of Bulk RNA-Seq Datasets Identifies Potential Biomarkers and Repurposable Therapeutics against Alzheimer’s Disease. Sci. Rep..

[2] Ashrafian H., Zadeh E.H., Khan R.H. (2021). Review on Alzheimer’s Disease: Inhibition of Amyloid Beta and Tau Tangle Formation. Int. J. Biol. Macromol..

[3] Alhazmi H.A., Albratty M. (2022). An Update on the Novel and Approved Drugs for Alzheimer Disease. Saudi Pharm. J..

[4] Dingezweni S. (2020). The Blood–Brain Barrier. South. Afr. J. Anaesth. Analg..

[5] Kam A., Li M., Razmovski-Naumovski V., Nammi S., Chan K., Li Y., Li Q. (2012). The Protective Effects of Natural Products on Blood-Brain Barrier Breakdown. Curr. Med. Chem..

[6] Nolan J.M., Power R., Howard A.N., Bergin P., Roche W., Prado-Cabrero A., Pope G., Cooke J., Power T., Mulcahy R. (2022). Supplementation With Carotenoids, Omega-3 Fatty Acids, and Vitamin E Has a Positive Effect on the Symptoms and Progression of Alzheimer’s Disease. J. Alzheimer’s Dis..

[7] Valdés A., Sánchez-Martínez J.D., Gallego R., Ibáñez E., Herrero M., Cifuentes A. (2024). In Vivo Neuroprotective Capacity of a *Dunaliella salina* Extract—Comprehensive Transcriptomics and Metabolomics Study. NPJ Sci. Food.

[8] Gallego R., Valdés A., Sánchez-Martínez J.D., Suárez-Montenegro Z.J., Ibáñez E., Cifuentes A., Herrero M. (2022). Study of the Potential Neuroprotective Effect of *Dunaliella salina* Extract in SH-SY5Y Cell Model. Anal. Bioanal. Chem..

[9] Iyer S., Bhat I., Bangera Sheshappa M. (2024). Lutein and the Underlying Neuroprotective Promise against Neurodegenerative Diseases. Mol. Nutr. Food Res..

[10] Medoro A., Davinelli S., Milella L., Willcox B.J., Allsopp R.C., Scapagnini G., Willcox D.C. (2023). Dietary Astaxanthin: A Promising Antioxidant and Anti-Inflammatory Agent for Brain Aging and Adult Neurogenesis. Mar. Drugs.

[11] Li Z., Cao Z., Chen F., Li B., Jin H. (2024). Lutein Inhibits Glutamate-Induced Apoptosis in HT22 Cells via the Nrf2/HO-1 Signaling Pathway. Front. Neurosci..

[12] Pruccoli L., Balducci M., Pagliarani B., Tarozzi A. (2024). Antioxidant and Neuroprotective Effects of Fucoxanthin and Its Metabolite Fucoxanthinol: A Comparative In Vitro Study. Curr. Issues Mol. Biol..

[13] Chen Y., Lu H., Ding Y., Liu S., Ding Y., Lu B., Xiao J., Zhou X. (2023). Dietary Protective Potential of Fucoxanthin as an Active Food Component on Neurological Disorders. J. Agric. Food Chem..

[14] Baek S.Y., Kim M.R. (2020). Neuroprotective Effect of Carotenoid-Rich Enteromorpha Prolifera Extract via TrkB/Akt Pathway against Oxidative Stress in Hippocampal Neuronal Cells. Mar. Drugs.

[15] Anbualakan K., Tajul Urus N.Q., Makpol S., Jamil A., Mohd Ramli E.S., Md Pauzi S.H., Muhammad N. (2023). A Scoping Review on the Effects of Carotenoids and Flavonoids on Skin Damage Due to Ultraviolet Radiation. Nutrients.

[16] Ikeda C., Manabe Y., Tomonaga N., Wada T., Maoka T., Sugawara T. (2020). Evaluation of Intestinal Absorption of Dietary Halocynthiaxanthin, a Carotenoid from the Sea Squirt *Halocynthia roretzi*. Mar. Drugs.

[17] Moran N.E., Mohn E.S., Hason N., Erdman J.W., Johnson E.J. (2018). Intrinsic and Extrinsic Factors Impacting Absorption, Metabolism, and Health Effects of Dietary Carotenoids. Adv. Nutr..

[18] Kobayashi Y., Sugahara K., Takemoto Y., Tsuda J., Hirose Y., Hashimoto M., Yamashita H. (2023). Protective Effect of Astaxanthin Nanoemulsion on Mammalian Inner Ear Hair Cells. PeerJ.

[19] Zanoni F., Vakarelova M., Zoccatelli G. (2019). Development and Characterization of Astaxanthin-Containing Whey Protein-Based Nanoparticles. Mar. Drugs.

[20] Freitas M.A., Ferreira J., Nunes M.C., Raymundo A. (2024). The Chemistry and Bioactive Properties behind Microalgae-Enriched Gluten-Free Breads. Int. J. Food Sci. Technol..

[21] Nunes M.C., Fernandes I., Vasco I., Sousa I., Raymundo A. (2020). *Tetraselmis chuii* as a Sustainable and Healthy Ingredient to Produce Gluten-Free Bread: Impact on Structure, Colour and Bioactivity. Foods.

[22] FDA (2016). GRAS Notice Inventory.

[23] Mantecón L., Moyano R., Cameán A.M., Jos A. (2019). Safety Assessment of a Lyophilized Biomass of *Tetraselmis chuii* (TetraSOD^®^) in a 90 Day Feeding Study. Food Chem. Toxicol..

[24] Conlon T., Aranyos A., Luck T., Touzet N. (2024). The Effects of Trophic Mode and Medium Composition on the Biochemical Profile and Antioxidant Capacity of *Tetraselmis chuii* (CCAP 66/21B). Biocatal. Agric. Biotechnol..

[25] Paterson S., Villanueva-Bermejo D., Hernández-Ledesma B., Gómez-Cortés P., de la Fuente M.A. (2024). Supercritical CO_2_ Extraction Increases the Recovery Levels of Omega-3 Fatty Acids in *Tetraselmis chuii* Extracts. Food Chem..

[26] Cocksedge S.P., Mantecón L., Castaño E., Infante C., Bailey S.J. (2025). The Potential of Superoxide Dismutase-Rich *Tetraselmis chuii* as a Promoter of Cellular Health. Int. J. Mol. Sci..

[27] Cokdinleyen M., Alvarez-Rivera G., Tejera J.L.G., Mendiola J.A., Valdés A., Kara H., Ibáñez E., Cifuentes A. (2024). *Tetraselmis chuii* Edible Microalga as a New Source of Neuroprotective Compounds Obtained Using Fast Biosolvent Extraction. Int. J. Mol. Sci..

[28] de Medeiros L.M., De Bastiani M.A., Rico E.P., Schonhofen P., Pfaffenseller B., Wollenhaupt-Aguiar B., Grun L., Barbé-Tuana F., Zimmer E.R., Castro M.A.A. (2019). Cholinergic Differentiation of Human Neuroblastoma SH-SY5Y Cell Line and Its Potential Use as an In Vitro Model for Alzheimer’s Disease Studies. Mol. Neurobiol..

[29] Isabel U.V., de la Riera M., Belén A., Dolores R.S., Elena G.B. (2024). A New Frontier in Neuropharmacology: Recent Progress in Natural Products Research for Blood–Brain Barrier Crossing. Curr. Res. Biotechnol..

[30] Sánchez-Martínez J.D., Garcia A.R., Alvarez-Rivera G., Valdés A., Brito M.A., Cifuentes A. (2023). In Vitro Study of the Blood–Brain Barrier Transport of Natural Compounds Recovered from Agrifood By-Products and Microalgae. Int. J. Mol. Sci..

[31] Lea T., Verhoeckx K. (2015). Caco-2 Cell Line. The Impact of Food Bioactives on Health: In Vitro and Ex Vivo Models.

[32] Cokdinleyen M., dos Santos L.C., de Andrade C.J., Kara H., Colás-Ruiz N.R., Ibañez E., Cifuentes A. (2024). A Narrative Review on the Neuroprotective Potential of Brown Macroalgae in Alzheimer’s Disease. Nutrients.

[33] Savvidou M.G., Georgiopoulou I., Antoniou N., Tzima S., Kontou M., Louli V., Fatouros C., Magoulas K., Kolisis F.N. (2023). Extracts from *Chlorella vulgaris* Protect Mesenchymal Stromal Cells from Oxidative Stress Induced by Hydrogen Peroxide. Plants.

[34] Guo G.X., Qiu Y.H., Liu Y., Yu L.L., Zhang X., Tsim K.W.K., Qin Q.W., Hu W.H. (2024). Fucoxanthin Attenuates Angiogenesis by Blocking the VEGFR2-Mediated Signaling Pathway through Binding the Vascular Endothelial Growth Factor. J. Agric. Food Chem..

[35] Dhapola R., Beura S.K., Sharma P., Singh S.K., HariKrishnaReddy D. (2024). Oxidative Stress in Alzheimer’s Disease: Current Knowledge of Signaling Pathways and Therapeutics. Mol. Biol. Rep..

[36] Pang Q.Q., Kim J.H., Kim H.Y., Kim J.H., Cho E.J. (2023). Protective effects and mechanisms of pectolinarin against H_2_O_2_-induced oxidative stress in SH-SY5Y neuronal cells. Molecules.

[37] Masters C.L., Bateman R., Blennow K., Rowe C.C., Sperling R.A., Cummings J.L. (2015). Alzheimer’s Disease. Nat. Rev. Dis. Primers.

[38] Manochkumar J., Doss C.G.P., El-Seedi H.R., Efferth T., Ramamoorthy S. (2021). The neuroprotective potential of carotenoids in vitro and in vivo. Phytomedicine.

[39] Meena N., Rout S., Mali S.N., Oliveira M., Campos Chisté R., Helena de Aguiar Andrade E., Santana de Oliveira M. (2024). Neuroprotective Potential of Carotenoids. Carotenoids.

[40] Banerjee S., Baghel D., Pacheco de Oliveira A., Ghosh A. (2023). β-Carotene, a Potent Amyloid Aggregation Inhibitor, Promotes Disordered Aβ Fibrillar Structure. Int. J. Mol. Sci..

[41] de Oliveira Caland R.B., Cadavid C.O.M., Carmona L., Peña L., de Paula Oliveira R. (2019). Pasteurized Orange Juice Rich in Carotenoids Protects *Caenorhabditis elegans* against Oxidative Stress and β-Amyloid Toxicity through Direct and Indirect Mechanisms. Oxid. Med. Cell. Longev..

[42] Brasil F.B., de Almeida F.J.S., Luckachaki M.D., Dall’Oglio E.L., de Oliveira M.R. (2021). Astaxanthin prevents mitochondrial impairment in the dopaminergic SH-SY5Y cell line exposed to glutamate-mediated excitotoxicity: Role for the Nrf2/HO-1/CO-BR axis. Eur. J. Pharmacol..

[43] Pap R., Pandur E., Jánosa G., Sipos K., Nagy T., Agócs A., Deli J. (2022). Lutein Decreases Inflammation and Oxidative Stress and Prevents Iron Accumulation and Lipid Peroxidation at Glutamate-Induced Neurotoxicity. Antioxidants.

[44] Inan B., Mutlu B., Cakır R., Balkanlı D. (2024). From ice to neurons: Investigating the neuroprotective effects of Antarctic microalgae *Chlorella variabilis* and *Chlorella pyrenoidosa* extracts. 3 Biotech..

[45] Park H.A., Hayden M.M., Bannerman S., Jansen J., Crowe-White K.M. (2020). Anti-Apoptotic Effects of Carotenoids in Neurodegeneration. Molecules.

[46] González-Peña M.A., Lozada-Ramírez J.D., Ortega-Regules A.E. (2021). Carotenoids from Mamey (*Pouteria sapota*) and Carrot (*Daucus carota*) Increase the Oxidative Stress Resistance of *Caenorhabditis elegans*. Biochem. Biophys. Rep..

[47] Shen J., Jiang C.Q., Yan Y.F., Liu B.R., Zu C.L. (2017). Effect of Increased UV-B Radiation on Carotenoid Accumulation and Total Antioxidant Capacity in Tobacco (*Nicotiana tabacum* L.) Leaves. Genet. Mol. Res..

[48] Wei J., Ye Z., Li Y., Li Y., Zhou Z. (2024). Citrus Carotenoid Extracts Promote ROS Accumulation and Induce Oxidative Stress to Exert Anti-Proliferative and Pro-Apoptotic Effects in MDA-MB-231 Cells. Antioxidants.

[49] Islam F., Khan J., Zehravi M., Das R., Haque M.A., Banu A., Parwaiz S., Nainu F., Nafady M.H., Shahriar S.M.S. (2024). Synergistic effects of carotenoids: Therapeutic benefits on human health. Process Biochem..

[50] Shi J., Kakuda Y., Yeung D. (2004). Antioxidative Properties of Lycopene and Other Carotenoids from Tomatoes: Synergistic Effects. Biofactors.

[51] Sowmya P., Arathi B., Vijay K., Baskaran V., Lakshminarayana R. (2017). Astaxanthin from Shrimp Efficiently Modulates Oxidative Stress and Allied Cell Death Progression in MCF-7 Cells Treated Synergistically with β-Carotene and Lutein from Greens. Food Chem. Toxicol..

[52] Chen X., Deng Z., Zheng L., Zhang B., Luo T., Li H. (2021). Interaction between Flavonoids and Carotenoids on Ameliorating Oxidative Stress and Cellular Uptake in Different Cells. Foods.

[53] Di L., Kerns E.H., Fan K., McConnell O.J., Carter G.T. (2003). High Throughput Artificial Membrane Permeability Assay for Blood–Brain Barrier. Eur. J. Med. Chem..

[54] Sánchez-Martínez J.D., Valdés A., Gallego R., Suárez-Montenegro Z.J., Alarcón M., Ibañez E., Alvarez-Rivera G., Cifuentes A. (2022). Blood–Brain Barrier Permeability Study of Potential Neuroprotective Compounds Recovered From Plants and Agri-Food by-Products. Front. Nutr..

[55] do Nascimento T.C., Pinheiro P.N., Fernandes A.S., Murador D.C., Neves B.V., de Menezes C.R., de Rosso V.V., Jacob-Lopes E., Zepka L.Q. (2021). Bioaccessibility and Intestinal Uptake of Carotenoids from Microalgae *Scenedesmus obliquus*. LWT.

[56] Cornelissen F.M.G., Markert G., Deutsch G., Antonara M., Faaij N., Bartelink I., Noske D., Vandertop W.P., Bender A., Westerman B.A. (2023). Explaining Blood-Brain Barrier Permeability of Small Molecules by Integrated Analysis of Different Transport Mechanisms. J. Med. Chem..

[57] Shah B., Dong X. (2022). Current Status of In Vitro Models of the Blood-Brain Barrier. Curr. Drug Deliv..

[58] Benz F., Liebner S. (2020). Structure and Function of the Blood–Brain Barrier (BBB). Handb. Exp. Pharmacol..

[59] Ávila-Gálvez M.Á., Marques D., Figueira I., Cankar K., Bosch D., Brito M.A., dos Santos C.N. (2023). Costunolide and Parthenolide: Novel Blood-Brain Barrier Permeable Sesquiterpene Lactones to Improve Barrier Tightness. Biomed. Pharmacother..

[60] Scalise A.A., Kakogiannos N., Zanardi F., Iannelli F., Giannotta M. (2021). The Blood–Brain and Gut–Vascular Barriers: From the Perspective of Claudins. Tissue Barriers.

[61] Hernandez L., Ward L.J., Arefin S., Ebert T., Laucyte-Cibulskiene A., Pilote L., Norris C.M., Raparelli V., Kautzky-Willer A., Herrero M.T. (2022). Blood–Brain Barrier and Gut Barrier Dysfunction in Chronic Kidney Disease with a Focus on Circulating Biomarkers and Tight Junction Proteins. Sci. Rep..

[62] Maria-Ferreira D., Nascimento A.M., Cipriani T.R., Santana-Filho A.P., Watanabe P.D.S., Sant’Ana D.D.M.G., Luciano F.B., Bocate K.C.P., van den Wijngaard R.M., Werner M.F.D.P. (2018). Rhamnogalacturonan, a Chemically-Defined Polysaccharide, Improves Intestinal Barrier Function in DSS-Induced Colitis in Mice and Human Caco-2 Cells. Sci. Rep..

[63] Amasheh M., Schlichter S., Amasheh S., Mankertz J., Zeitz M., Fromm M., Schulzke J.D. (2008). Quercetin Enhances Epithelial Barrier Function and Increases Claudin-4 Expression in Caco-2 Cells. J. Nutr..

[64] Iftikhar M., Iftikhar A., Zhang H., Gong L., Wang J. (2020). Transport, Metabolism and Remedial Potential of Functional Food Extracts (FFEs) in Caco-2 Cells Monolayer: A Review. Food Res. Int..

[65] Failla M.L., Rodrigues D.B., Chitchumroonchokchai C., Mercadante A. (2019). Bioavailability and Metabolism of Carotenoid Esters. Carotenoid Esters in Foods: Physical, Chemical and Biological Properties.

[66] Flieger J.F.A.F.W. (2024). Carotenoid Supplementation for Alleviating the Symptoms of Alzheimer’s Disease. Int. J. Mol. Sci..

[67] Si P., Zhu C. (2022). Biological and Neurological Activities of Astaxanthin (Review). Mol. Med. Rep..

[68] Galasso C., Orefice I., Pellone P., Cirino P., Miele R., Ianora A., Brunet C., Sansone C. (2018). On the Neuroprotective Role of Astaxanthin: New Perspectives?. Mar. Drugs.

[69] Paul R., Mazumder M.K., Nath J., Deb S., Paul S., Bhattacharya P., Borah A. (2020). Lycopene—A Pleiotropic Neuroprotective Nutraceutical: Deciphering Its Therapeutic Potentials in Broad Spectrum Neurological Disorders. Neurochem. Int..

[70] Yang M., Xuan Z., Wang Q., Yan S., Zhou D., Naman C.B., Zhang J., He S., Yan X., Cui W. (2022). Fucoxanthin Has Potential for Therapeutic Efficacy in Neurodegenerative Disorders by Acting on Multiple Targets. Nutr. Neurosci..

[71] Sekiya M., Suzuki S., Ushida Y., Sato I., Suganuma H. (2023). Neoxanthin Is Undetectable in Human Blood after Ingestion of Fresh Young Spinach Leaf. PLoS ONE.

[72] Tang G.F., Zhang M.R., Liu Q.Q., Mai R.R. (2022). Applications of nanodiamonds in the diagnosis and treatment of neurological diseases. J. Nanopart. Res..

[73] Lopalco A., Cutrignelli A., Denora N., Lopedota A., Franco M., Laquintana V. (2018). Transferrin Functionalized Liposomes Loading Dopamine HCl: Development and Permeability Studies across an In Vitro Model of Human Blood-Brain Barrier. Nanomaterials.

[74] Rehman A., Tong Q., Jafari S.M., Assadpour E., Shehzad Q., Aadil R.M., Iqbal M.W., Rashed M.M.A., Mushtaq B.S., Ashraf W. (2020). Carotenoid-loaded nanocarriers: A comprehensive review. Adv. Colloid. Interface Sci..

[75] Huang S., Ding X. (2022). Precise Design Strategies of Nanotechnologies for Controlled Drug Delivery. J. Funct. Biomater..

[76] Shitara Y., Maeda K., Ikejiri K., Yoshida K., Horie T., Sugiyama Y. (2013). Clinical Significance of Organic Anion Transporting Polypeptides (OATPs) in Drug Disposition: Their Roles in Hepatic Clearance and Intestinal Absorption. Biopharm. Drug Dispos..

[77] Lin J.K., Chen P.C., Ho C.T., Lin-Shiau S.Y. (2000). Inhibition of Xanthine Oxidase and Suppression of Intracellular Reactive Oxygen Species in HL-60 Cells by Theaflavin-3,3′-Digallate, (-)- Epigallocatechin-3-Gallate, and Propyl Gallate. J. Agric. Food Chem..

[78] Pogačnik L., Pirc K., Palmela I., Skrt M., Kwang K.S., Brites D., Brito M.A., Ulrih N.P., Silva R.F.M. (2016). Potential for Brain Accessibility and Analysis of Stability of Selected Flavonoids in Relation to Neuroprotection in Vitro. Brain Res..

[79] Deli M.A., Ábrahám C.S., Kataoka Y., Niwa M. (2005). Permeability Studies on in Vitro Blood-Brain Barrier Models: Physiology, Pathology, and Pharmacology. Cell. Mol. Neurobiol..

[80] Rojo-Poveda O., Barbosa-Pereira L., Khattabi C.E., Youl E.N.H., Bertolino M., Delporte C., Pochet S., Stévigny C. (2020). Polyphenolic and Methylxanthine Bioaccessibility of Cocoa Bean Shell Functional Biscuits: Metabolomics Approach and Intestinal Permeability through CaCo-2 Cell Models. Antioxidants.

