# Supplementary material for: Neuroprotective Potential of Tetraselmis chuii Compounds: Insights into Blood–Brain Barrier Permeability and Intestinal Transport
Source: Pharmaceuticals (Basel). 2025 Apr 26;18(5):629. doi: 10.3390/ph18050629 (PMC12115197; doi:10.3390/ph18050629)
Supplement: Supplementary file 1 [file pharmaceuticals-18-00629-s001.zip › pharmaceuticals-3566247-supplementary.pdf]

Supplementary Figures

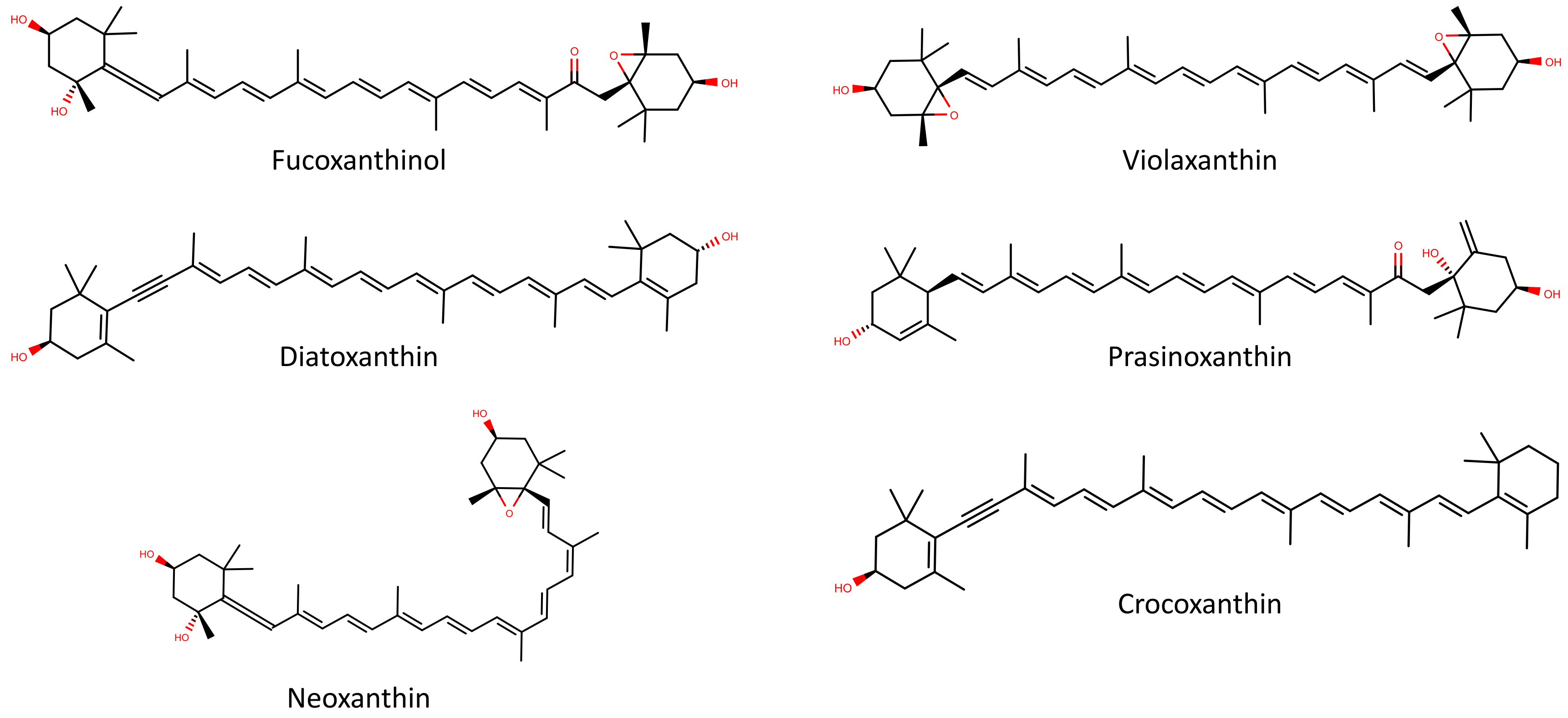

Figure S1. Chemical structures of quantified carotenoids.

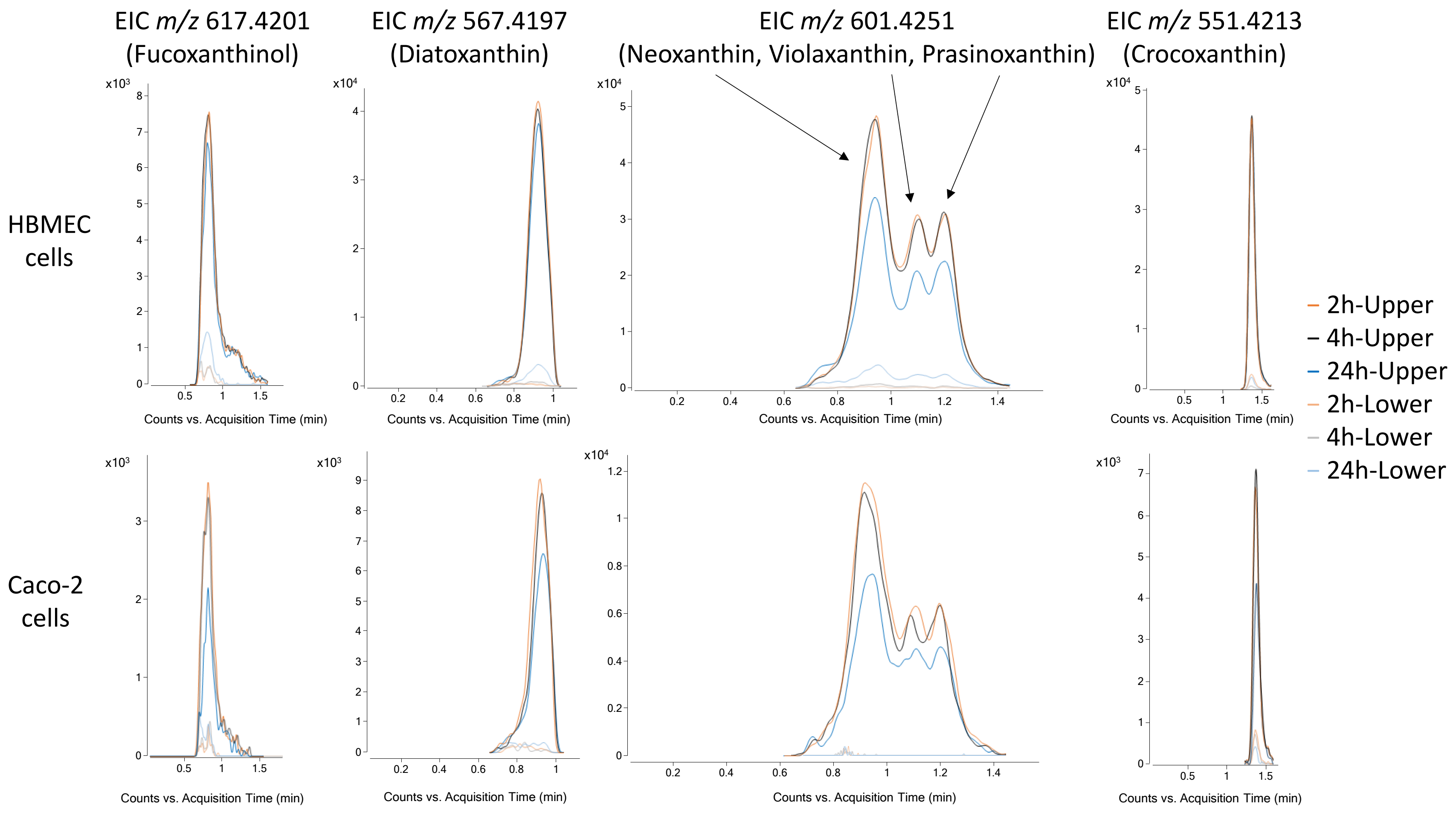

Figure S2. Extracted Ion Chromatograms (EIC) of quantified carotenoids in HBMEC and Caco-2 cells, after different incubation times (2h, 4h and 24h).
